# Effect of altitude and acetazolamide on postural control in healthy lowlanders 40 years of age or older. Randomized, placebo-controlled trial

**DOI:** 10.3389/fphys.2023.1274111

**Published:** 2024-01-04

**Authors:** Tim Mutschler, Michael Furian, Mona Lichtblau, Aline Buergin, Simon R. Schneider, Paula Appenzeller, Laura Mayer, Lara Muralt, Maamed Mademilov, Ainura Abdyraeva, Shoira Aidaralieva, Aibermet Muratbekova, Azamat Akylbekov, Saltanat Shabykeeva, Talant M. Sooronbaev, Silvia Ulrich, Konrad E. Bloch

**Affiliations:** ^1^ Department of Respiratory Medicine, University Hospital Zurich, Zurich, Switzerland; ^2^ Swiss-Kyrgyz High Altitude Medicine and Research Initiative, Zurich, Switzerland; ^3^ Swiss-Kyrgyz High Altitude Medicine and Research Initiative, Bishkek, Kyrgyzstan; ^4^ Department of Respiratory Medicine, National Center of Cardiology and Internal Medicine, Bishkek, Kyrgyzstan

**Keywords:** altitude (MeSH), hypoxia, postural control, age, altitude-related adverse health effects, altitude illness, prevention, acetazolamide

## Abstract

**Background:** Hypoxia and old age impair postural control and may therefore enhance the risk of accidents. We investigated whether acetazolamide, the recommended drug for prevention of acute mountain sickness, may prevent altitude-induced deterioration of postural control in older persons.

**Methods:** In this parallel-design trial, 95 healthy volunteers, 40 years of age or older, living <1,000 m, were randomized to preventive therapy with acetazolamide (375 mg/d) or placebo starting 24 h before and during a 2-day sojourn at 3,100 m. Instability of postural control was quantified by a balance platform with the center of pressure path length (COPL) as primary outcome while pulse oximetry (SpO_2_) was monitored. Effects of altitude and treatment on COPL were evaluated by ordered logistic regression. www.ClinicalTrials.gov NCT03536429.

**Results:** In participants taking placebo, ascent from 760 m to 3,100 m increased median COPL from 25.8 cm to 27.6 cm (odds ratio 3.80, 95%CI 2.53–5.70) and decreased SpO_2_ from 96% to 91% (odds ratio 0.0003, 95%CI 0.0002–0.0007); in participants taking acetazolamide, altitude ascent increased COPL from 24.6 cm to 27.3 cm (odds ratio 2.22, 95%CI 1.57–3.13), while SpO_2_ decreased from 96% to 93% (odds ratio 0.007, 95%CI 0.004–0.012). Altitude-induced increases in COPL were smaller with acetazolamide vs. placebo (odds ratio 0.58, 95%CI 0.34–0.99) while drops in SpO_2_ were mitigated (odds ratio 19.2, 95%CI 9.9–37.6).

**Conclusion:** In healthy individuals, 40 years of age or older, postural control was impaired after spending a night at 3,100 m. The altitude-induced deterioration of postural control was mitigated by acetazolamide, most likely due to the associated improvement in oxygenation.

## Introduction

Travel to high mountain regions is increasingly popular. Modern means of transportation allow rapid ascent to high altitudes even for untrained and older people, a growing proportion of mountain tourists. Exposure to hypobaric hypoxia at high altitude may cause adverse health effects including altitude illness (Furian et al., 2022), poor sleep (Latshang et al., 2013), psychomotor and learning impairment (Scheiwiller et al., 2022; Reiser et al., 2023) and deterioration of postural control (Stadelmann et al., 2015; Buergin et al., 2023). The latter is of particular concern as it may lead to potentially dangerous falls. Adequate postural control is the result of a complex process involving the vestibular, visual, somatosensory systems, as well as central processing and a rapid and precise response of the musculoskeletal system to any perturbances (Yoshida et al., 1988; Berry et al., 1989; Delliaux and Jammes, 2006). At high altitude, postural control is mainly impaired by hypobaric hypoxia. Acetazolamide (AZA), a drug used for prevention and treatment of acute mountain sickness (AMS) (Luks et al., 2019) is a carbonic anhydrase inhibitor which induces a metabolic acidosis via increased renal bicarbonate excretion and, thus, stimulates ventilation (Adamson and Swenson, 2017). This improves arterial oxygenation and prevents the occurrence of periodic breathing and AMS at high altitude (Graf et al., 2023). Carbonic anhydrase is also present in nervous tissue and its inhibition by AZA has been found to reduce cerebral oxygen consumption and improve the cerebral tissue oxygenation under hypoxic conditions (Wang et al., 2015).

The aim of the current randomized, placebo-controlled trial was therefore to corroborate the impairment of postural control in healthy individuals travelling to high altitude and to test the hypothesis that administration of AZA prevents the altitude-related impairment of postural control. We focused on older persons as it has been shown that advanced age is associated with worsening postural control (Era et al., 2006), in particular at high altitude (Bruyneel et al., 2017).

## Materials and methods

### Study design and setting

This study was part of a randomized, placebo-controlled, double-blinded trial evaluating the efficacy of preventive AZA treatment in reducing AMS in healthy lowlanders 40 years of age or older during a 2-day sojourn at 3,100 m (Too Ashu High Altitude Clinic, Kyrgyz Republic). Data on effects of altitude and AZA on AMS and physiologic outcomes have been reported elsewhere (Furian et al., 2022). Data on postural control, the focus of the current study, have not been published. The study was approved by the Ethics Committee of the National Center of Cardiology and Internal Medicine, Bishkek, Kyrgyz Republic (2018-10) and was registered at ClinicalTrials.gov NCT03536429. Participants gave written informed consent.

### Participants

Healthy men and women, 40 years of age or older, living in Bishkek or nearby regions (Kyrgyz Republic, mean altitude 760 m) were invited to participate. Inclusion criteria were age of 40–75 years, living at a location <1,000 m and good health without any active disease.

### Interventions

Participants underwent baseline measurements at the National Center for Cardiology and Internal Medicine in Bishkek at 760 m a few days before ascent by minibus within 3–5 h to the Too Ashu High Altitude Clinic at 3,100 m where they stayed for 2 days and nights. Supervised study medication intake started 1 day before and continued during the stay at high altitude. Participants received AZA or equally looking placebo capsules, 125 mg in the morning and 250 mg in the evening (total dose 375 mg daily). As in a similar study in patients with chronic obstructive pulmonary disease (Buergin et al., 2023), we choose to administer a higher dose of AZA in the evening to prevent sleep-related hypoventilation and a lower dose in the morning to avoid excessive stimulation of ventilation with dyspnea (Furian et al., 2022).

### Assessments

At both altitudes, a medical history, clinical examination and pulse oximetry (SpO_2_, Minolta PULSOX- 300i) were obtained.

The Lake Louise questionnaire was used to assess the presence and severity of AMS. It assesses the subjectively experienced severity of 5 different symptom complexes. The scale ranges from 0 to 15 points. A score of 3 or more, including headache, is considered to indicate the presence of AMS (Roach et al., 1993).

**FIGURE 1:**
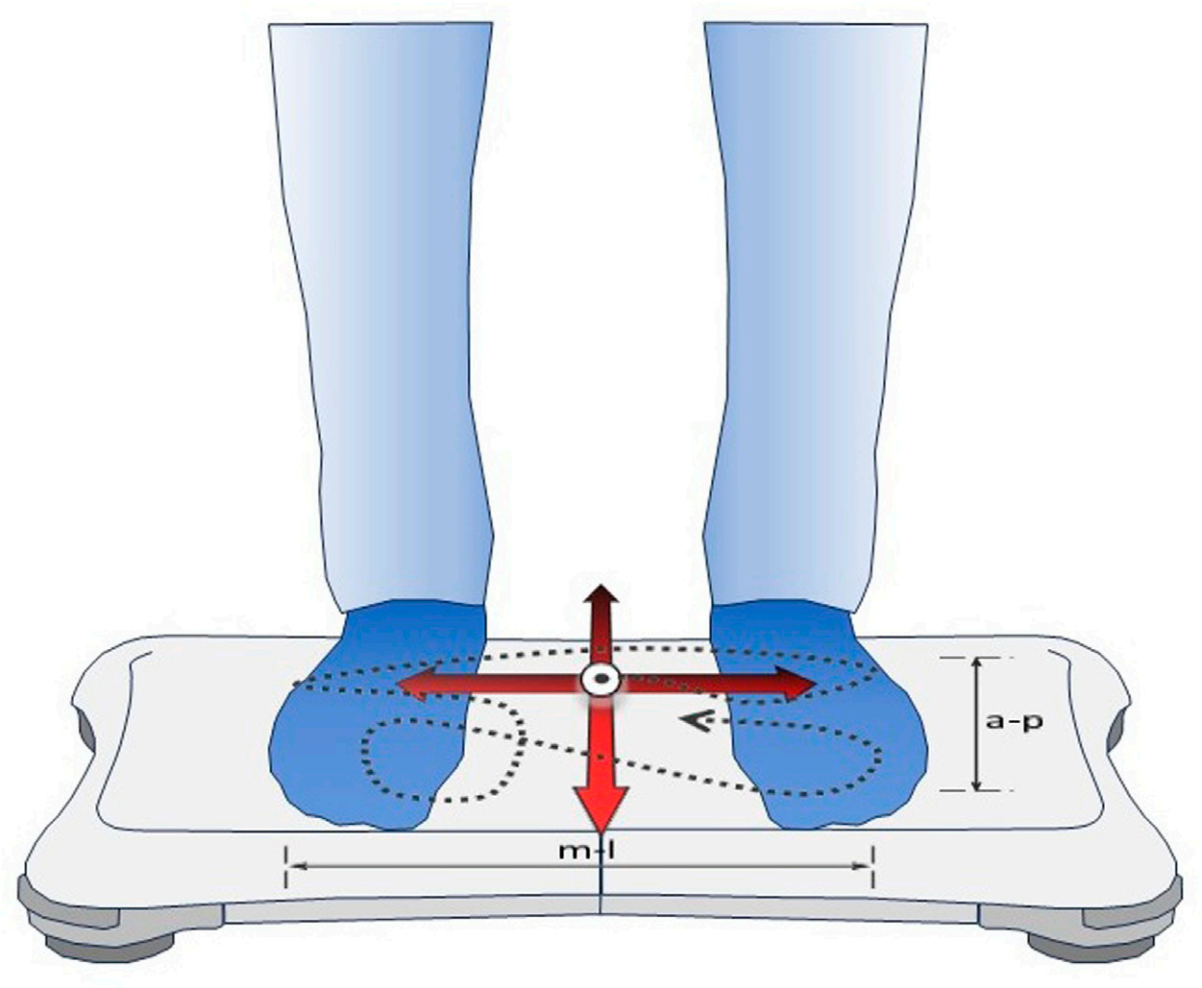
Assessment of postural control by the balance platform. The commercially available Wii Balance Board (Nintendo, Kyoto, Japan) used built-in sensors to record trajectories of the center of pressure (dashed line) generated by the subject during quiet standing on both feet. Custom-built software computed the center of pressure path length (COPL, the length of the dashed line) and sway amplitudes and velocities in antero-posterior (a-p) and medio-lateral (m-l) directions during 30-s trials.

Baseline evaluation of postural control was performed at 760 m, before study drug intake, and at 760 m, after the third dose (i.e., after a total dose of 500 mg), approximately 24 h after the first study drug intake, to assess the effect of AZA in normobaric, normoxic conditions. Further evaluations were performed in the morning after one night at 3,100 m. A balance platform (Wii Balance Board, Nintendo, Kyoto, Japan), a validated tool for assessing standing balance (Clark et al., 2010), was employed as described previously and illustrated in Figure 1 (Stadelmann et al., 2015; Muralt et al., 2018; Buergin et al., 2023). Participants performed a series of five 30-s trials of quiet standing with open eyes and both feet on the balance board with 2–3 min rest intervals. They were instructed to position their feet in a 30° angle, to keep arms beside the body and to look at a 2 cm black spot, placed on a wall at eye level at a distance of 1.5 m. A customized software (Labview 8.5 National Instruments, Austin, TX, United States) was used to calculate the primary outcome, the center of pressure path length (COPL), as well as sway velocity and amplitude in antero-posterior (AP) and medio-lateral (ML) directions.

### Randomization and blinding

Participants were randomly assigned to AZA or placebo treatment with a 1:1 allocation as per computer generated schedule minimizing for age (<50, 50-59 and 60–75 years) and sex (MinimPy 0.3, Distributed under the GNU GPL v3) (Saghaei, 2011).

Identically looking AZA and placebo capsules, labelled with secret codes, were prepared by an independent pharmacist. Blinding of investigators and participants was maintained until completion of data analysis.

### Data analysis

The primary analysis was performed on the per-protocol population including all participants who had successful evaluations at both altitudes. To account for non-normal distribution, data were summarized by medians and quartiles. Additionally, analysis of the primary outcome, the COPL, was also performed on the intention-to-treat population defined as all randomized participants. Missing data were replaced by multiple imputations (n = 20) (White et al., 2011). The effects of altitude and medication were evaluated by ordered logistic regression on quintiles of outcomes (i.e., COPL and other indices of postural control) with the study drug as independent variable. The analyses were adjusted for sex, age, and height since older age and larger height are known to be associated with larger COPL (Muralt et al., 2018). A probability of *p* < 0.05 or a 95% confidence interval (CI) not overlapping an odds ratio of 1 in logistic regression analysis were considered statistically significant.

## Results

**FIGURE 2:**
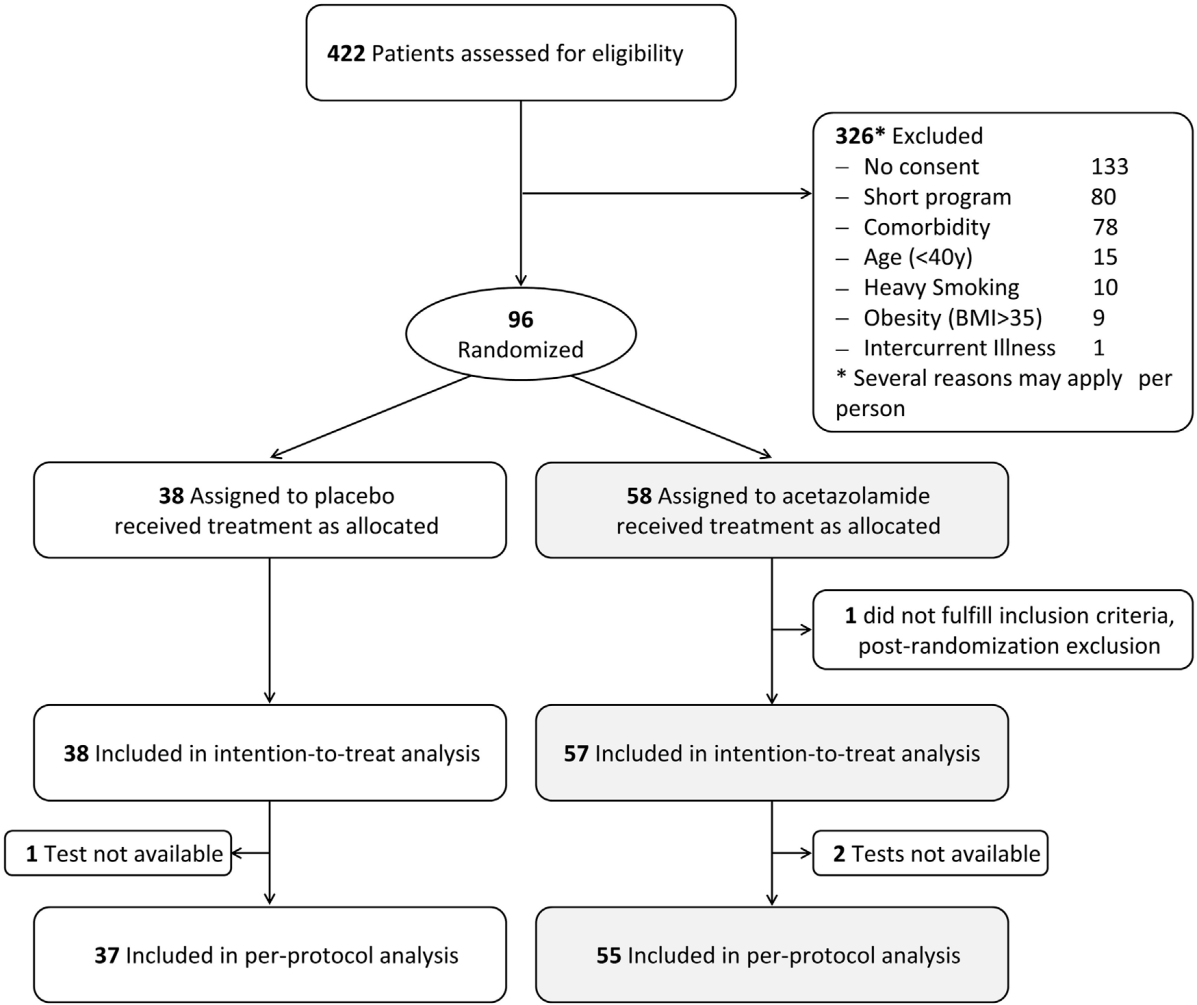
Participant flow.

Ninety-five participants fulfilled the inclusion criteria and were included in the intention-to-treat analysis. In 1 participant in the placebo group and 2 participants in the AZA group, balance tests were not available at both altitudes for various reasons. Therefore, per protocol analysis was performed with the data of 92 participants (Figure 2). Demographic characteristics are outlined in Table 1. Participants had a median (quartiles) age of 53.6 (49.4, 57.4) y and a body mass index (BMI) of 27.1 (24.6, 30.1) kg/m^2^.

**TABLE 1:** Participants characteristics–per protocol population at 760 m.

	All	Placebo	Acetazolamide
N, (% female)	92 (59%)	37 (56%)	55 (62%)
Age, years	53.6 (49.4, 57.4)	52.9 (48.0, 57.2)	54.2 (50.1, 58.2)
Weight, kg	70.5 (62.8, 77.2)	71.6 (63.3, 79.1)	68.9 (62.8, 76.9)
Height, m	1.61 (1.57, 1.68)	1.62 (1.57, 1.68)	1.61 (1.55, 1.68)
Body mass index, kg/m^2^	27.1 (24.6, 30.1)	27.2 (24.3, 29.7)	27.1 (24.6, 30.5)

Medians (quartiles).

AMS occurred in a total of 14 of 38 (37%) participants receiving placebo and in 11 of 57 (19%) receiving AZA (*p* < 0.05). Among these, 4 participants each from the placebo (11%) and AZA (7%) groups suffered from AMS at the time of assessment of postural control (morning after the first night at 3,100 m).

### Altitude effect

**FIGURE 3:**
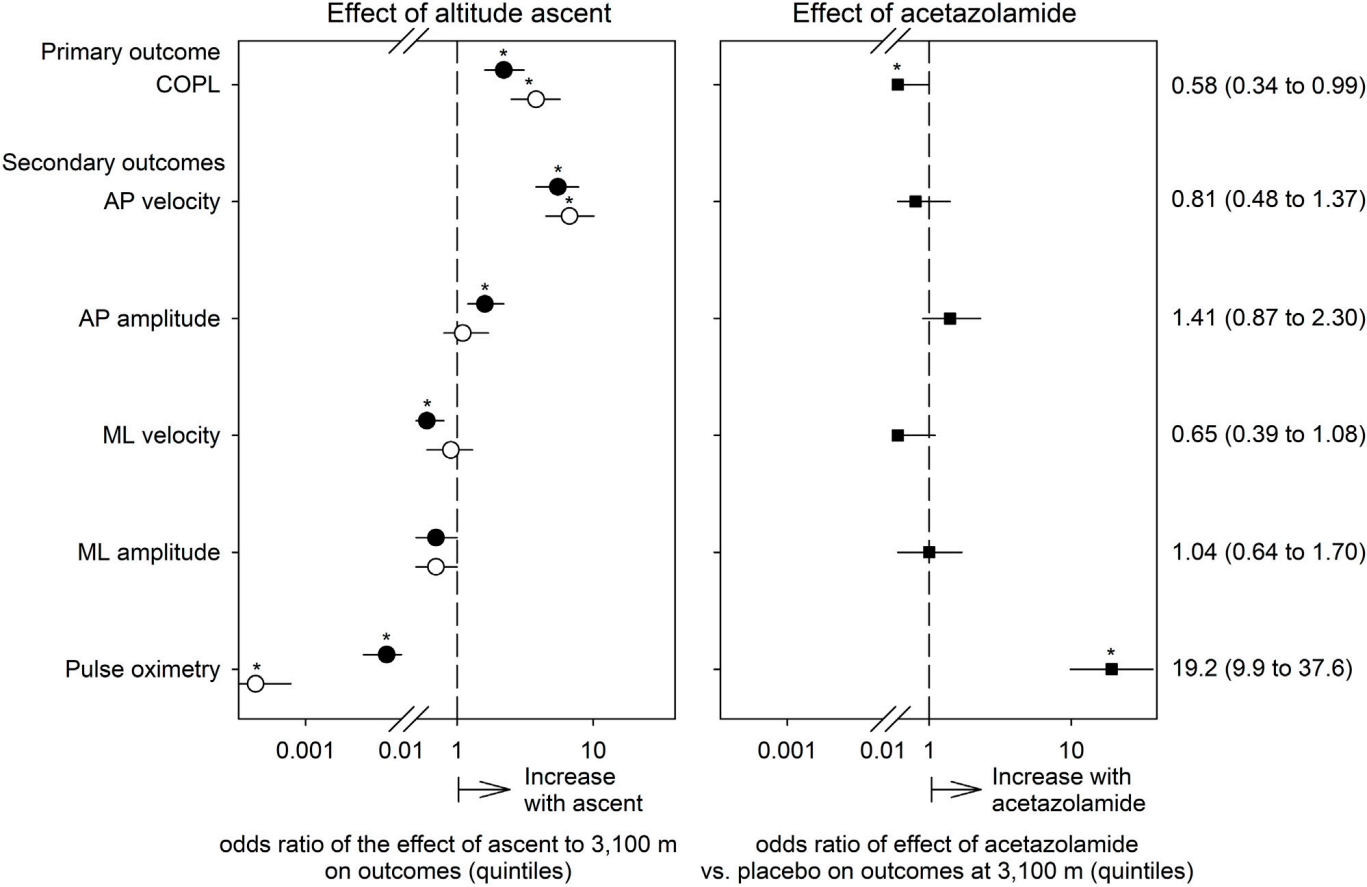
Effects of ascent to high altitude (left panel) and of preventive acetazolamide treatment (right panel) on indices of postural control and pulse oximetry evaluated by ordered logistic regression. The dependent variables in these analyses were quintiles of the outcomes. Thus, the odds ratios (symbols) with their 95% confidence intervals (lines) displayed on a logarithmic x-axis scale reflect the probability of a change in outcome from one to the next higher quintile in relation to no such increase (i.e., p/[1-p]). The odds ratios are adjusted for the effects of sex, age and body height. Open circles represent the altitude-effect in the placebo group, closed circles the altitude-effect in the acetazolamide group, and squares the effects of acetazolamide vs. placebo at 3,100 m. Asterisks mark significant effects (95% confidence intervals not overlapping 1, *p* < 0.05).

In the per-protocol population, the COPL in the group treated with placebo increased with ascent from 760 to 3,100 m from a median of 25.8 to 27.6 cm. In the AZA group, the altitude-induced increase in median COPL was from 24.6 cm to 27.3 cm. These changes were statistically significant (Table 2; Figure 3). The altitude-induced COPL changes in the intention-to-treat population were similar (data not shown).

**TABLE 2:** Indices of postural control and pulse oximetry at 760 m and 3,100 m in the groups assigned to acetazolamide and placebo treatment.

	Placebo			Acetazolamide		
	760 m without medication	760 m with medication	3,100 m with medication	760 m without medication	760 m with medication	3,100 m with medication
Center of pressure path length, cm	24.9 (20.5; 29.6)	25.8 (21.7; 29.6)	27.6 (23.1; 31.7)**	25.2 (21.6; 31.0)	24.6 (21.4; 30.9)	27.3 (23.3; 32.8)**
Antero-posterior sway velocity; cm/s	0.6 (0.5; 0.7)	0.6 (0.5; 0.7)	0.7 (0.6; 0.8)**	0.6 (0.5; 0.8)	0.6 (0.5; 0.7)	0.7 (0.6; 0.8)**
Antero-posterior sway amplitude, cm	2.1 (1.8; 2.7)	2.1 (1.7; 2.6)	2.2 (1.8; 2.7)	2.1 (1.7; 2.6)	2.1 (1.8; 2.6)	2.3 (1.8; 2.8)**
Medio-lateral sway velocity; cm/s	0.5 (0.4; 0.5)	0.5 (0.4; 0.5)	0.5 (0.4; 0.5)	0.5 (0.4; 0.5)	0.4 (0.4; 0.5)	0.4 (0.4; 0.5)**
Medio-lateral sway amplitude, cm	1.5 (1.2, 1.9)	1.3 (1.1, 1.8)**	1.4 (1.2, 1.7)	1.6 (1.2, 2)	1.5 (1.1, 1.8)	1.4 (1.1, 1.9)
Pulse oximetry, %	96 (96, 97)	96 (95, 97)	91 (90, 92)**	96 (95, 97)	97 (95, 97)	93 (91, 93)**

Medians (quartiles) of data from the per protocol population, n = 92.

*p < 0.05, **p < 0.01, Wilcoxon matched pairs test within respective group vs. baseline with respective medication at 760 m, i.e., with placebo or acetazolamide, respectively.

AP sway velocity increased significantly with the ascent from 760 to 3,100 m in both groups whereas the ML sway velocity did not change in the placebo group but decreased in the AZA group (Table 2; Figure 3). AP sway amplitude did not change in the placebo group but increased significantly in the AZA group. ML sway amplitude did not significantly change with ascent to 3,100 m in any group (Figure 3).

### Effect of acetazolamide on postural control

The altitude-induced increase in COPL in the AZA group was significantly smaller than in the placebo group when adjusted for age, sex, height (Table 3; Figure 3). No significant effects of AZA on the altitude-induced changes of AP and ML sway velocity and amplitude were observed (Tables 4A, B; Figure 3).

**TABLE 3:** Effects of high altitude and acetazolamide on the primary outcome, the center of pressure path length. Results of ordered logistic regression analysis.

Dependent variable: Quintiles of the center of pressure path length (COPL)					
Predictors		Odds ratio	SE	95% CI	*p*-value
Placebo group	760 m, without medication	1	NA	NA	NA
	760 m, with placebo	1.30	0.26	0.87 to 1.93	0.204
	3,100 m, with placebo*	3.79	0.78	2.53 to 5.67	<0.001
Acetazolamide vs. placebo group	760 m, without medication	0.98	0.27	0.58 to 1.67	0.945
	760 m, with acetazolamide	0.64	0.17	0.38 to 1.09	0.101
	3,100 m, with acetazolamide**	0.56	0.15	0.33 to 0.94	0.030
Age, years		1.01	0.02	0.98 to 1.05	0.452
Men vs. women		0.47	0.15	0.25 to 0.90	0.022
Height, m		0.64	1.20	0.02 to 25.61	0.810
Intercept, center of pressure path length (COPL), cm		1.44	0.03	1.38 to 1.51	<0.001

The analysis was performed on data of the per protocol population, n = 92.

* altitude effect in the placebo group.

** treatment effect of acetazolamide vs. placebo at 3,100 m.

**TABLE 4:** Effects of high altitude and acetazolamide on the secondary outcomes. Results of ordered logistic regression analysis.

A: Dependent variable: Quintiles of the antero-posterior sway velocity						
Predictors			Odds ratio	SE	95% CI	*p*-value
Placebo group		760 m, without medication	1	NA	NA	NA
		760 m, with placebo	1.50	0.31	1.01 to 2.24	0.044
		3,100 m, with placebo*	6.76	1.40	4.51 to 10.14	<0.001
Acetazolamide vs. placebo group		760 m, without medication	0.96	0.27	0.55 to 1.68	0.886
		760 m, with acetazolamide	0.76	0.20	0.45 to 1.29	0.316
		3,100 m, with acetazolamide**	0.78	0.21	0.46 to 1.31	0.348
Age, years			1.04	0.02	1.00 to 1.01	0.044
Men vs. women			0.42	0.15	0.21 to 0.84	0.015
Height, m			0.57	1.15	0.01 to 30.85	0.780
Intercept, antero-posterior sway velocity, cm/s			1.42	0.03	1.35 to 1.48	<0.001
B: Dependent variable: quintiles of the medio-lateral sway velocity						
Predictors			Odds ratio	SE	95% CI	P
Placebo group	760 m, without medication		1	NA	NA	NA
	760 m, with placebo		0.96	0.19	0.65 to 1.42	0.843
	3,100 m, with placebo*		0.90	0.18	0.61 to 1.33	0.599
Acetazolamide vs. placebo group	760 m, without medication		0.79	0.28	0.39 to 1.60	0.512
	760 m, with acetazolamide		0.81	0.21	0.48 to 1.36	0.427
	3,100 m, with acetazolamide**		0.63	0.17	0.38 to 1.05	0.079
Age, years			0.97	0.02	0.92 to 1.02	0.191
Men vs. Women			0.57	0.27	0.22 to 1.44	0.234
Height, m			0.79	2.17	0.00 to 171.05	0.932
Intercept, medio-lateral sway velocity at 760 m, cm/s			1.33	0.04	1.26 to 1.41	<0.001

The analysis was performed on data of the per protocol population, n = 92.

* altitude effect in the placebo group. ** treatment effect of acetazolamide vs. placebo at 3,100 m.

To evaluate any effects of AZA on postural control independent of the effect of hypoxia, measurements at 760 m before and after starting the drug treatment were performed. There were no significant effects of AZA on COPL nor on AP nor ML sway velocity (Tables 3, 4).

## Discussion

The current randomized, placebo controlled double blind trial in healthy individuals 40 years of age or older shows that postural control is impaired after ascent from 760 m to 3,100 m as demonstrated by an increase in COPL and antero-posterior sway velocity measured on a balance platform. The altitude-induced prolongation in COPL was mitigated by preventive acetazolamide treatment in association with improved oxygenation compared to placebo.

The altitude-induced deterioration of postural control observed in the current study is consistent with observations in previous studies in younger healthy individuals and in patients with chronic obstructive pulmonary disease (Stadelmann et al., 2015; Clarke et al., 2018; Muralt et al., 2018). These data suggest that the reduced oxygen partial pressure at high altitude and the resulting hypoxia is causative for the impaired postural control even though the detailed mechanisms are unknown.

In the current study, the altitude-induced alteration in postural control was mainly reflected in a longer COPL that was related to an increased AP sway velocity and amplitude. These observations are consistent with previous studies, which also showed that altitude exposure increased the sway in anterior-posterior direction (Muralt et al., 2018; Buergin et al., 2023). It has been suggested that this may be due to the anatomical alignment of the leg and feet joints that result in a greater flexibility in the antero-posterior compared to the medio-lateral direction and to the greater sensitivity of the visual corrective input to lateral vs. antero-posterior alterations (Nordahl et al., 1998). Furthermore, the elevated respiratory rate resulting from hypoxia at high altitude may cause more movements in antero-posterior direction (Hodges et al., 2002).

Although the minimally clinically important difference in COPL and other measures of postural control is not established we assume that any altitude-induced deterioration in postural control may enhance the risk of potentially dangerous falls and mitigation of this altitude-related adverse effect by AZA seems therefore desirable.

As there is an increasing proportion of older persons among altitude travelers, we have studied individuals 40 years of age and older. Our data suggest that older age within this range had a negative effect on AP sway velocity while the COPL and medio-lateral sway velocity were not associated with age (Tables 3, 4). A worse postural control of older compared to younger mountaineers has been reported previously (Bruyneel et al., 2017; Buergin et al., 2023). Moreover, a large-scale study has shown that postural control deteriorates with increasing age even at sea level (Era et al., 2006). Although our data confirm an association of age with impairment of postural control in hypoxic conditions our conclusions cannot be extrapolated to individuals below the age of 40 years.

Eleven percent of participants in the placebo group and 7% in the AZA group suffered from AMS at the time of postural control assessment. AMS may lead to dizziness, light-headedness and ataxia (Roach et al., 1993). Because only a small proportion of our participants suffered from AMS, the potential effect of AMS on postural control independent of the effects of hypoxia *per se* could not be conclusively assessed. Nevertheless, previous studies have suggested that postural control can deteriorate independently of the presence of AMS (Baumgartner and Bartsch, 2002; Baumgartner et al., 2002; Clarke et al., 2018; Muralt et al., 2018). This is conceivable given the subjective nature of AMS diagnosis compared to the quantitative assessment of postural control by a balance platform. Importantly, this discrepancy may have clinical implications as they suggest that an individual may not subjectively appreciate impairment in postural control during exposure to hypoxia.

In our trial AZA mitigated the increase in COPL at high altitude although the effect seems to be modest. In a previous study performed in patients with chronic obstructive pulmonary disease of similar age as the participants in the current study, postural control was also impaired at altitude but this was not prevented by AZA treatment (Buergin et al., 2023).

We found that 500 mg AZA taken in 3 doses (125/250/125 mg) in intervals of about 12 h over the course of approximately 24 h, had no effect on postural control at 760 m. This is reassuring as Collier et al. (Collier et al., 2016) observed that intake of a single dose of 500 mg AZA resulted in impaired postural control at sea level. The absence of an adverse effect of acetazolamide on postural control near sea level in the current study might be due to the lower single doses of 125 mg and 250 mg administered over the course of approximately 24 h.

A major strength of our trial is to provide robust evidence of altitude-related deterioration of postural control in a large group of older individuals and to show that AZA mitigates this adverse effect of hypobaric hypoxia. This study was part of a larger trial investigating the efficacy of preventive AZA treatment in reducing AMS (Furian et al., 2022) and participants underwent various assessments other than just the balance board measurements including exercise tests which may have affected performance in postural control due to muscular fatigue (Paillard, 2012). Since postural control was assessed before exercise tests and because the same tests were performed at both altitudes and in both groups, it is unlikely that the various assessments have affected the conclusions.

In conclusion, postural control was impaired in healthy individuals aged 40 years or older after the first night at 3,100 m compared to 760 m. This effect was reduced by the preventive intake of AZA starting 24 h before the ascent. Since AZA also reduced AMS (Furian et al., 2022), a preventive treatment with AZA should be considered in elderly subjects travelling to high altitude in order to reduce the risk of suffering from altitude-related adverse health effects including the potential risk of falls due to impaired postural control.

## Data Availability

The raw data supporting the conclusion of this article will be made available by the authors, without undue reservation.

## References

[B1] Adamson R. Swenson E. R. (2017). Acetazolamide use in severe chronic obstructive pulmonary disease. Pros and cons. Ann. Am. Thorac. Soc. 14 (7), 1086–1093. 10.1513/AnnalsATS.201701-016FR 28622013

[B2] Baumgartner R. W. Bartsch P. (2002). Ataxia in acute mountain sickness does not improve with short-term oxygen inhalation. High. Alt. Med. Biol. 3 (3), 283–287. 10.1089/152702902320604269 12396882

[B3] Baumgartner R. W. Eichenberger U. Bartsch P. (2002). Postural ataxia at high altitude is not related to mild to moderate acute mountain sickness. Eur. J. Appl. Physiol. 86 (4), 322–326. 10.1007/s00421-001-0534-8 11990745

[B4] Berry D. T. McConnell J. W. Phillips B. A. Carswell C. M. Lamb D. G. Prine B. C. (1989). Isocapnic hypoxemia and neuropsychological functioning. J. Clin. Exp. Neuropsychol. 11 (2), 241–251. 10.1080/01688638908400886 2494222

[B5] Bruyneel A. V. Humbert A. Bertrand M. (2017). Comparison of balance strategies in mountain climbers during real altitude exposure between 1.500m and 3.200m: effects of age and expertise. Neurosci. Lett. 657, 16–21. 10.1016/j.neulet.2017.06.010 28743580

[B6] Buergin A. Furian M. Mayer L. Lichtblau M. Scheiwiller P. M. Sheraliev U. (2023). Effect of acetazolamide on postural control in patients with COPD travelling to 3100 m randomized trial. J. Clin. Med. 12 (4), 1246. 10.3390/jcm12041246 36835782 PMC9960941

[B7] Clark R. A. Bryant A. L. Pua Y. McCrory P. Bennell K. Hunt M. (2010). Validity and reliability of the Nintendo Wii balance board for assessment of standing balance. Gait Posture 31 (3), 307–310. 10.1016/j.gaitpost.2009.11.012 20005112

[B8] Clarke S. B. Deighton K. Newman C. Nicholson G. Gallagher L. Boos C. J. (2018). Changes in balance and joint position sense during a 12-day high altitude trek: the British Services Dhaulagiri medical research expedition. PLoS One 13 (1), e0190919. 10.1371/journal.pone.0190919 29342191 PMC5771604

[B9] Collier D. J. Wolff C. B. Hedges A. M. Nathan J. Flower R. J. Milledge J. S. (2016). Benzolamide improves oxygenation and reduces acute mountain sickness during a high-altitude trek and has fewer side effects than acetazolamide at sea level. Pharmacol. Res. Perspect. 4 (3), e00203. 10.1002/prp2.203 27433337 PMC4876137

[B10] Delliaux S. Jammes Y. (2006). Effects of hypoxia on muscle response to tendon vibration in humans. Muscle Nerve 34 (6), 754–761. 10.1002/mus.20633 16941658

[B11] Era P. Sainio P. Koskinen S. Haavisto P. Vaara M. Aromaa A. (2006). Postural balance in a random sample of 7,979 subjects aged 30 years and over. Gerontology 52 (4), 204–213. 10.1159/000093652 16849863

[B12] Furian M. Buergin A. Schweiwiller P. M. Mayer L. Schneider S. Emilov B. (2022). Acetazolamide to prevent adverse altitude effects in COPD and healthy adults. NEJM Evid. 1 (1). 10.1056/evidoa2100006 38296630

[B13] Graf L. C. Furian M. Bitos K. Mademilov M. Abdraeva A. Buenzli J. (2023). Effect of altitude and acetazolamide on sleep and nocturnal breathing in healthy lowlanders 40y of age or older. Data from a randomized trial. Sleep 46, zsac269. 10.1093/sleep/zsac269 36356042

[B14] Hodges P. W. Gurfinkel V. S. Brumagne S. Smith T. C. Cordo P. C. (2002). Coexistence of stability and mobility in postural control: evidence from postural compensation for respiration. Exp. Brain Res. 144 (3), 293–302. 10.1007/s00221-002-1040-x 12021811

[B15] Latshang T. D. Lo Cascio C. M. Stowhas A. C. Grimm M. Stadelmann K. Tesler N. (2013). Are nocturnal breathing, sleep, and cognitive performance impaired at moderate altitude (1,630-2,590 m)? Sleep 36 (12), 1969–1976. 10.5665/sleep.3242 24293773 PMC3825448

[B16] Luks A. M. Auerbach P. S. Freer L. Grissom C. K. Keyes L. E. McIntosh S. E. (2019). Wilderness medical society clinical practice guidelines for the prevention and treatment of acute altitude illness: 2019 update. Wilderness Environ. Med. 30, S3–S18. 10.1016/j.wem.2019.04.006 31248818

[B17] Muralt L. Furian M. Lichtblau M. Aeschbacher S. S. Clark R. A. Estebesova B. (2018). Postural control in lowlanders with COPD traveling to 3100 m: data from a randomized trial evaluating the effect of preventive dexamethasone treatment. Front. Physiol. 9, 752. 10.3389/fphys.2018.00752 29988503 PMC6024910

[B18] Nordahl S. H. Aasen T. Owe J. O. Molvaer O. I. (1998). Effects of hypobaric hypoxia on postural control. Aviat. Space Environ. Med. 69 (6), 590–595. 9641406

[B19] Paillard T. (2012). Effects of general and local fatigue on postural control: a review. Neurosci. Biobehav Rev. 36 (1), 162–176. 10.1016/j.neubiorev.2011.05.009 21645543

[B20] Reiser A. E. Furian M. Lichtblau M. Buergin A. Schneider S. R. Appenzeller P. (2023). Effect of acetazolamide on visuomotor performance at high altitude in healthy people 40 years of age or older-RCT. PLoS One 18 (1), e0280585. 10.1371/journal.pone.0280585 36662903 PMC9858039

[B21] Roach R. C. Bartsch P. Hackett P. H. Oelz O. (1993). “The Lake Louise acute montain sickness scoring system,” in Hypoxia and molecular medicine: proceedings of the 8th international hypoxia symposium. Editors Sutton J. R. Huston C. S. Coates G. (Burlington: Queen City Press), p772–p774.

[B22] Saghaei M. (2011). An overview of randomization and minimization programs for randomized clinical trials. J. Med. Signals Sens. 1 (1), 55–61. 10.4103/2228-7477.83520 22606659 PMC3317766

[B23] Scheiwiller P. M. Furian M. Buergin A. Mayer L. C. Schneider S. R. Mademilov M. (2022). Visuomotor performance at high altitude in COPD patients. Randomized placebo-controlled trial of acetazolamide. Front. Physiol. 13, 980755. 10.3389/fphys.2022.980755 36160864 PMC9493049

[B24] Stadelmann K. Latshang T. D. Lo Cascio C. M. Clark R. A. Huber R. Kohler M. (2015). Impaired postural control in healthy men at moderate altitude (1630 m and 2590 m): data from a randomized trial. PLoS One 10 (2), e0116695. 10.1371/journal.pone.0116695 25723529 PMC4344242

[B25] Wang K. Smith Z. M. Buxton R. B. Swenson E. R. Dubowitz D. J. (2015). Acetazolamide during acute hypoxia improves tissue oxygenation in the human brain. J. Appl. Physiol. (1985) 119 (12), 1494–1500. 10.1152/japplphysiol.00117.2015 26472861 PMC4683345

[B26] White I. R. Royston P. Wood A. M. (2011). Multiple imputation using chained equations: issues and guidance for practice. Stat. Med. 30 (4), 377–399. 10.1002/sim.4067 21225900

[B27] Yoshida S. Sasa M. Takaori S. (1988). Different sensitivity to hypoxia in neuronal activities of lateral vestibular and spinal trigeminal nuclei. Stroke 19 (3), 357–364. 10.1161/01.str.19.3.357 2895509

